# Acute histoplasmosis in four immunocompetent Canadian travellers to a cenote in Yucatán, Mexico

**DOI:** 10.14745/ccdr.v51i05a05

**Published:** 2025-05-01

**Authors:** Elspeth MacBain, Michael Hawkes, David Goldfarb, Jan Hajek

**Affiliations:** 1 Department of Pediatrics, University of British Columbia, Vancouver, BC; 2 Department of Pathology and Laboratory Medicine, University of British Columbia, Vancouver, BC; 3 Department of Infectious Diseases, University of British Columbia, Vancouver, BC

**Keywords:** histoplasmosis, travel medicine, fever in returned traveler, endemic fungi

## Abstract

A group of four healthy Canadian travellers visited a cenote in the Yucatán peninsula in April 2024 and subsequently developed symptomatic histoplasmosis. Diagnosis was made in the acute period with a positive urine *Histoplasma* antigen test in three of the cases. Two developed severe presentations and were treated with itraconazole, including a three-year-old child with disseminated disease. The sensitivity of different modalities for diagnostics depends on the timing and severity of illness, with *Histoplasma* urine antigen being most sensitive in early infection, serology converting 4–8 weeks following exposure and cultures generally of low sensitivity. Treatment depends on the disease manifestations and host immunologic status. Many patients have relatively mild, self-limited, influenza-like illness and the diagnosis may be overlooked. Given the number of Canadian tourists travelling to the Yucatán peninsula and the popularity of visiting cenotes, awareness of the risk of histoplasmosis associated with this exposure should be promoted.

## Introduction

Histoplasmosis is a fungal infection caused by inhalation of the microconidia of *Histoplasma capsulatum*. It is endemic in various regions across the world and is classically associated with exposure to bat and bird excrement. In Canada, *Histoplasma* is endemic along the St. Lawrence Seaway in Ontario and Québec and recent case reports have described local acquisition in Alberta and Saskatchewan ((1)). *Histoplasma* is well known to be regionally endemic in the Ohio and Mississippi River Valleys of central and eastern United States and common in Mexico, Central and South America, several regions in Africa and South East Asia ((1)).

Infection is often asymptomatic, with population seroprevalence in some endemic areas as high as 80% ((2)); however, histoplasmosis can have a spectrum of clinical manifestations ranging from mild self-limited influenza-like-illness, to severe pneumonia and disseminated disease ((3)). Symptoms typically begin 1–3 weeks following exposure ((4)). Young children, immune-compromised hosts and the elderly are at higher risk of severe disease. A number of cases of histoplasmosis have been described in immunocompetent travellers to endemic areas, often in association with exposure to bat-caves ((5)). Histoplasmosis is not a nationally notifiable disease in Canada, so the true incidence of infection nationally is unknown and it is possible that many cases go and/or unreported.

## Case series

In April 2024, four (three confirmed, one suspected) Canadian travellers, two 37-year-old males, a 36-year-old female and a three-year-old male, developed symptomatic histoplasmosis after swimming in Cenote Aktunzots near Tres Reyes in the Yucatán peninsula of Mexico. They recalled seeing bats inside the cenote. Two other members of their party, a 33-year-old woman and a two-year-old male, stayed at the resort rather than attending the cenote and remained asymptomatic. Patient consent was obtained to share the following information.

### Case 1

**Figure 1:**
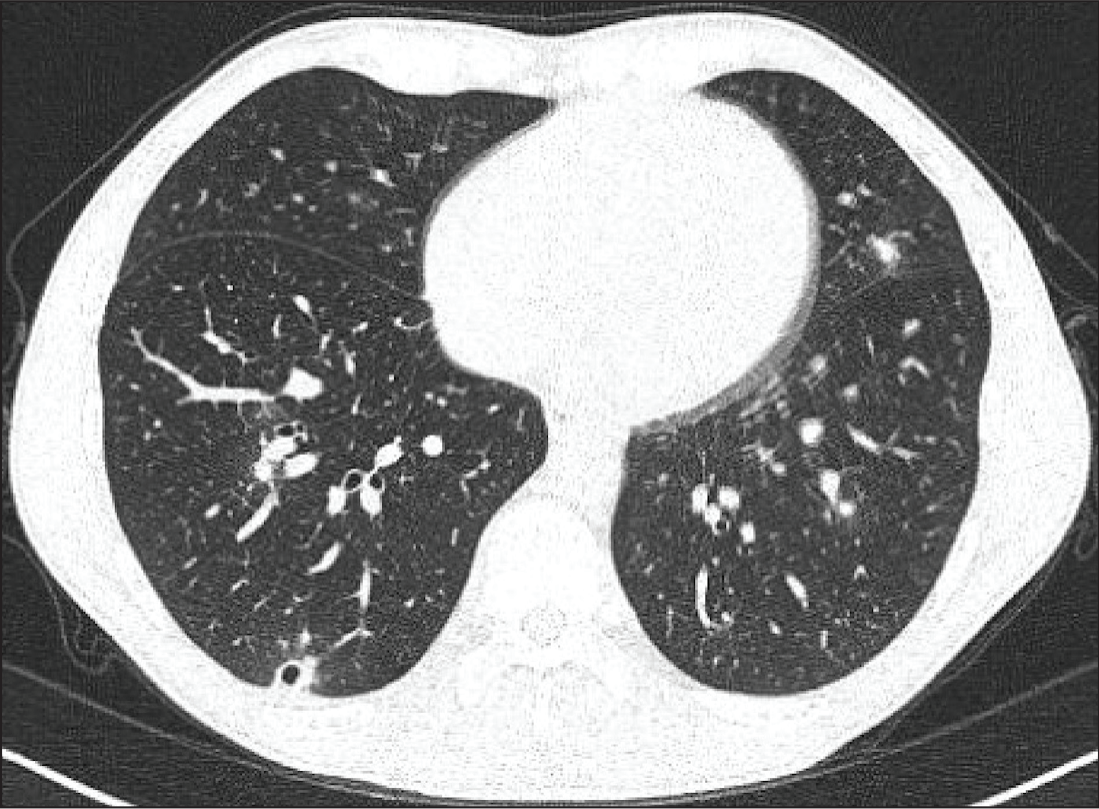
Computed tomography of chest of Case 1, histoplasmosis in Canadian traveller, 2025

Approximately 18 days after visiting the cenote, the 37-year-old male developed fevers, myalgia, fatigue and then cough. He was found to have bilateral pulmonary nodules with cavitation and ground-glass opacities on computed tomography scan (**Figure 1**) and underwent bronchoalveolar lavage on day 14 of illness. Bacterial, fungal and mycobacterial bronchoalveolar lavage cultures were negative. Leptospirosis, dengue, syphilis and HIV serologies were negative. At day 11 of illness *H. capsulatum* serology was negative, but urine *Histoplasma* antigen was positive (MiraVista Diagnostics, Indianapolis, Indiana).

Because of ongoing flu-like symptoms significantly impairing his daily function, itraconazole was started on day 14 of the illness. He received a total of six weeks of itraconazole. Following treatment, his fevers abated and symptoms improved. He did not receive antibiotics. A repeat computed tomography scan six weeks after treatment discontinuation showed resolution of the cavitating nodule and reduction of bilateral nodules and ground-glass opacities. *Histoplasma capsulatum* serology repeated 10 weeks after the initial diagnosis was positive.

### Case 2

Two days after Case 1 developed symptoms, his son, a previously healthy three-year-old male developed daily high-grade fevers (39°C), myalgias, abdominal pain and headaches. At time of initial assessment, he had been symptomatic for 10 days. On examination, he was noted to have an oral aphthous ulcer, several small erythematous papules on his trunk, cervical and inguinal lymphadenopathy and hepatomegaly. Alanine aminotransferase was mildly elevated at 58 U/L (normal 10–29 U/L) and lactate dehydrogenase was elevated at 585 U/L (normal 207–383 U/L). Chest X-ray revealed bilateral hilar lymphadenopathy, fine nodularity and trace pleural effusions in the bases. Abdominal ultrasound showed mild hepatomegaly with relatively increased periportal echoes (“starry sky appearance”), a bulky spleen and intra-abdominal adenopathy. *Histoplasma capsulatum* serology was initially negative and urine was positive for *Histoplasma* antigen. Human immunodeficiency virus serology was negative.

Itraconazole, dosed at 10 mg/kg/day divided BID, was initiated on day 17 of illness. His fevers resolved within 48 hours of treatment initiation and his energy quickly improved. He received three months total duration of itraconazole. Therapeutic drug monitoring was done two weeks after itraconazole initiation and monthly afterward, targeting levels between 1,420 nmol/L and 4,840 nmol/L. The patient’s itraconazole level was initially in target range, then the dose was decreased to 7.5 mg/kg/day approximately six weeks into therapy, after the repeat level was elevated. He did not experience any adverse medication effects. Repeat *H. capsulatum* serology drawn approximately eight weeks after the initial diagnosis was positive. Prior to discontinuing treatment at three months, a repeat abdominal ultrasound demonstrated resolution of previous findings and reported as a normal study. Repeat chest X-rays showed interval resolution of the perihilar thickening, decreased fine nodularity bilaterally, with residual perihilar adenopathy. A chest X-ray three months post treatment discontinuation was normal.

### Case 3

Several days following symptom onset of Case 1, approximately three weeks following the visit to the cenote, the other 37-year-old male developed a self-limited febrile illness with myalgia and fatigue but without respiratory symptoms. His symptoms lasted approximately 12 days. Urine *Histoplasma* antigen was positive and initial *H. capsulatum* serology was negative. Human immunodeficiency virus serology was negative. Due to absence of respiratory symptoms, no chest imaging was done. No antifungal therapy was prescribed and he made a full recovery.

### Case 4

The 36-year-old female also developed cough and flu-like illness upon return to Canada, just over two weeks from exposure to the cenote. She did not have chest imaging, but was diagnosed clinically with probable community-acquired pneumonia by her family physician and was prescribed amoxicillin. Her symptoms were relatively mild and gradually resolved over the next 1–2 weeks. She did not receive antifungal therapy. Urine *Histoplasma* antigen and histoplasma serology performed approximately three weeks after her symptom onset were negative. Repeat convalescent serology was not available.

## Discussion

Cenotes (sinkholes) of the Yucatán Peninsula are flooded caves that are a popular tourist attraction, drawing crowds of local and international visitors ((6)); however, these cenotes have previously been implicated in exposure to *H. capsulatum*. The federal Ministry of Health of Mexico declared an outbreak between July and August 2022 after five tourists were diagnosed with histoplasmosis at a local hospital after visiting a cenote located in the municipality of Homún ((7)). Another cenote in same region has been closed since 2019 due to association with cases of histoplasmosis among tourists ((8)).

The high attack rate observed in this group of exposed travellers, with two developing more severe disease, suggests they likely encountered high concentrations of *H. capsulatum* spores at the cenote. On the other hand, the relatively long period of time between exposure to symptom onset (approximately three weeks, on the upper end of the typical 1–3 week incubation period), may be suggestive of a lower inoculum.

The symptoms of histoplasmosis can be non-specific and may lead to delayed diagnosis and unnecessary invasive diagnostic procedures ((9)). The sensitivity and specificity of testing modalities depends on the patient’s clinical syndrome, host-immune factors, timing and type of specimen collection. In general, *Histoplasma* antigen testing (e.g., MiraVista Diagnostics, Indianapolis, Indiana) is considered the most sensitive test in acute illness, but may miss milder infections with lower fungal burden in immunocompetent hosts ((10)).

Of our group, three individuals tested positive for urine *Histoplasma* antigen. Both of our cases who presented with more severe illness were initiated on empiric treatment while waiting for these test results. Our fourth case presented with symptoms of a non-specific influenza-like illness, suggestive of acute pulmonary histoplasmosis because of the epidemiologic context. The diagnosis was not confirmed by available laboratory testing; urine antigen testing was negative. The sensitivity of urine antigen testing is estimated to be 80% in acute pulmonary histoplasmosis, 30% in subacute pulmonary and 90% in progressive disseminated histoplasmosis ((10)).

All four cases had negative initial *H. capsulatum* serologies drawn approximately 2–4 weeks after exposure. In Cases 1 and 2, serologies that were repeated after 8–10 weeks turned positive. Unfortunately, repeat serologies in Cases 3 and 4 were not obtained. Histoplasma antibody testing was performed via immunodiffusion by the Alberta Provincial Laboratory for Public Health and included detection of both H and M antibodies ((11)). *Histoplasma capsulatum* antibodies typically take 4–8 weeks to become detectable in peripheral blood. Serology is estimated to have a sensitivity of approximately 65% in acute pulmonary histoplasmosis, 95% in subacute pulmonary, 83% in chronic pulmonary and 75% in progressive disseminated histoplasmosis. Antibody testing is most useful in subacute and chronic forms of histoplasmosis where sensitivity of urine antigen is decreased ((10)). Fungal cultures of the one patient who underwent bronchoalveolar lavage were ultimately negative. Culture-based methods can be challenging for informing acute management as growth typically takes 2–3 weeks and may only be positive in the minority of patients with acute pulmonary histoplasmosis (0%–20%) ((10)).

Recent guidelines suggest testing for histoplasmosis in all patients with community-acquired pneumonia without improvement on empiric antibiotics and exposure to an endemic area, or on initial presentation of community-acquired pneumonia in patients with high risk exposure to bird or bat droppings, or epidemiologic link to histoplasmosis outbreak ((12)). Practitioners should be aware that local acquisition outside the Great Lakes region is also possible in Canada ((11)). The urine *Histoplasma* antigen test is recommended as first line, with *H. capsulatum* serology more useful for subacute or chronic presentations ((12)).

## Conclusion

Given the number of Canadian tourists travelling to the Yucatán peninsula and the increasing popularity of visiting cenotes, awareness of the risk associated with this specific exposure should be promoted. Cenote exposure should raise the index of suspicion for histoplasmosis in symptomatic returning travellers.
